# Direct-Acting Antivirals in Hepatitis C Treatment for Renal Impairment: Liver Safety Concerns and Effectiveness in Peritoneal Dialysis

**DOI:** 10.3390/biomedicines13010055

**Published:** 2024-12-29

**Authors:** Hsuan-Yu Hung, Wei-Liang Hung, Ye Gu, Chung-Yu Chen

**Affiliations:** 1 School of Pharmacy, College of Pharmacy, Kaohsiung Medical University, Kaohsiung 80708, Taiwan; ameeyo36@gmail.com; 2 Department of Pharmacy, Ditmanson Medical Foundation Chia-Yi Christian Hospital, Chiayi 60002, Taiwan; 3 Division of Nephrology, Department of Medicine, Zuoying Armed Forces General Hospital, Kaohsiung 813204, Taiwan; lydership@gmail.com; 4 Graduate Institute of Natural Products, College of Pharmacy, Kaohsiung Medical University, Kaohsiung 80708, Taiwan; quye3000@hotmail.com; 5 Master Program in Clinical Pharmacy, School of Pharmacy, Kaohsiung Medical University, Kaohsiung 80708, Taiwan; 6 Department of Medical Research, Kaohsiung Medical University Hospital, Kaohsiung 80708, Taiwan; 7 Department of Pharmacy, Kaohsiung Medical University Hospital, Kaohsiung 80708, Taiwan

**Keywords:** drug-induced liver injury, DILI, liver damage, elbasvir/grazoprevir, glecaprevir/pibrentasvir, chronic kidney disease, chronic hepatitis C, HCV, direct-acting antivirals, peritoneal dialysis, PD

## Abstract

**Background/Objectives:** Glecaprevir/pibrentasvir (G/P) and elbasvir/grazoprevir (EBR/GZR) are effective treatments for chronic hepatitis C (CHC), especially in patients with chronic kidney disease (CKD). However, both regimens carry a risk of drug-induced liver injury (DILI). This study investigates the association between renal failure and DILI, using real-world data, and assesses the effectiveness of these treatments in peritoneal dialysis patients. **Methods:** A retrospective cohort study was conducted using data from the Ditmanson Research Database, including patients with CHC treated with G/P or EBR/GZR from 1 August 2017 to 31 December 2020. Patients were categorized into CKD and normal kidney function (NKF) groups. Two sensitivity analyses were performed to assess DILI risk. The study was approved by the DMF-CYCH Institutional Review Board (CYCH IRB No.: 2021010). **Results:** In 837 patients, DILI risk, expressed as incidence rate ratios (IRR), was 0.64 (95% CI 0.25–1.63) in the NKF group and 1.29 (95% CI 0.12–14.23) in the CKD group. Sensitivity analyses showed consistent results. A case–time–control analysis suggested liver instability despite treatment, with comorbid liver tumors (aOR 18.89; 95% CI 5.4–66.12) and hypertension (aOR 4.25; 95% CI 1.49–12.15) linked to higher DILI risk. All peritoneal dialysis patients (*n* = 10) achieved a 100% SVR12 rate. **Conclusions:** This real-world study supports the effectiveness of G/P and EBR/GZR in peritoneal dialysis patients. Comorbidities that impair liver function are key predictors of abnormal liver parameters, highlighting the need for careful monitoring during CHC treatment.

## 1. Introduction

Hepatitis C virus (HCV) infection is still a massive global health burden, with progression to chronic liver disease, cirrhosis, and hepatocellular carcinoma leading to considerable morbidity and mortality worldwide [1,2]. The evolution of HCV management has been revolutionized by the advent of direct-acting antivirals (DAAs), which have sustained virological response (SVR) rates greater than 95% and a better side effect profile compared to older interferon-based regimens [3,4]. Such developments have been especially successful in patients suffering from chronic kidney disease (CKD), which is associated with an increased burden of HCV stemming from both greater exposure to blood products, as well as dialysis-related procedures and nosocomial infestations and so forth.

The prevalence of hepatitis C is significantly higher among dialysis-dependent CKD patients than in the general population. This is in part due to the reuse of dialysis needles and equipment, as well as poor sterilization practices, which are significant routes of transmission [5]. HCV remains a crucial healthcare concern in Taiwan, where the prevalence of dialysis therapy among patients is extremely high worldwide [6]. Moreover, we found only a few studies published on the safety and efficacy of DAAs in patients currently on peritoneal dialysis, which is a specific group of CKD stage 5 patients that inevitably have to manage differently [4]. The U.S. FDA has identified these risks in safety alerts about the hepatotoxicity of G/P and EBR/GZR in patients with moderate to severe liver impairment, leading to the recommendation of close monitoring in such vulnerable populations [6,7].

Despite the efficacy of DAAs like glecaprevir/pibrentasvir (G/P) and elbasvir/grazoprevir (EBR/GZR) to treat HCV in many populations, drug-induced liver injury (DILI) remains a concern. Acute DILI, which is defined as either hepatocellular or cholestatic liver injury, occurs through a variety of mechanisms, including mitochondrial dysfunction, oxidative stress, and immune-mediated hepatotoxicity. Fatty liver disease is further exacerbated in patients with CKD already impaired drug clearance and altered pharmacokinetics leading to increased sensitivity to hepatotoxic metabolites [8,9].

Clinical guidelines, including those from the American Association for the Study of Liver Diseases and the Infectious Diseases Society of America (AASLD-IDSA), currently recommend G/P and EBR/GZR as appropriate regimens for HCV patients with stage 4–5 CKD, including dialysis patients [3]. Nonetheless, the majority of recommendations are derived from the data of patients undergoing hemodialysis, creating a knowledge gap pertaining to the implementation of DAAs in the peritoneal dialysis population. Peritoneal dialysis has some advantages over hemodialysis, such as better residual renal function preservation and a lower risk of bloodstream infection [2,3,4].

This report seeks to address these knowledge gaps using two retrospective pharmacovigilance-based studies to evaluate the association of renal impairment with the risk of DILI in patients receiving G/P or EBR/GZR. It also evaluates the efficacy of these regimens in eliciting SVR, particularly in patients on peritoneal dialysis. Utilizing population-based data from Taiwan, an area with the dual burden of HCV and CKD, this study aims to elucidate meaningful interpretations of antiviral therapy for this neglected disease cohort.

## 2. Materials and Methods

### 2.1. Study Design

This retrospective cohort study was conducted in southern Taiwan, using patient medical records from the Ditmanson Research Database (DRD) spanning from 1 August 2017 to 31 December 2020. The DRD is a continually updated repository containing comprehensive demographic, clinical, pharmacological, and laboratory data. Participants were divided into two groups: those with CKD and those with normal kidney function (NKF). The follow-up period was extended to 31 March 2021, with the index date incorporated into the analysis.

Due to the observational nature of the study and the anonymization of the data, the requirement for written informed consent was waived in accordance with the Human Subjects Research Act, as reviewed and approved by the Institutional Review Board of the Ditmanson Medical Foundation Chia-Yi Christian Hospital (DMF-CYCH) (CYCH IRB No.: 2021010).

### 2.2. Patient Population

**Figure 1:**
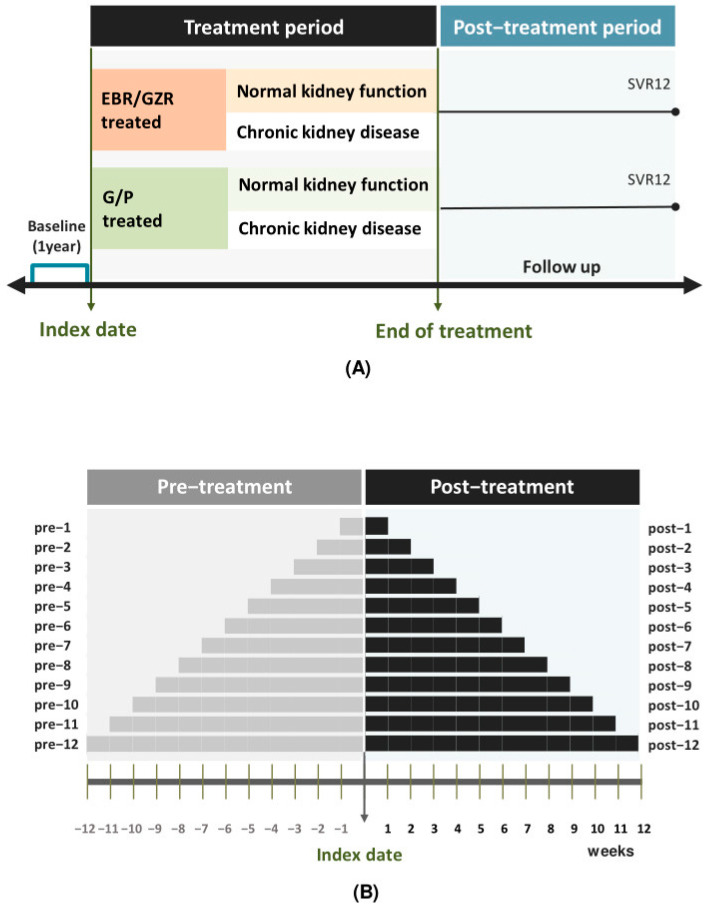
Flowchart of study population selection and study scheme and definitions of treatment periods. (**A**) study procedures (**B**) the case–time–control design diagram and definitions of treatment periods.

Inclusion criteria required patients to be 18 years or older, diagnosed with chronic hepatitis C (CHC) between 1 August 2017 and 31 December 2020, and treated with either the G/P (100 mg/40 mg) or EBR/GZR (100 mg/50 mg) regimen at DRD. Patients receiving EBR/GZR were classified as the reference group, while those treated with G/P were considered the intervention group. The choice of treatment regimen and the addition of Ribavirin (RBV) were determined by the patient’s overall health and the severity of cirrhosis. A one-year baseline period was utilized to assess baseline covariates, with comorbidities identified according to the International Classification of Diseases. The cohort was divided into two groups for renal function comparison: a CKD group (patients diagnosed with CKD or those with an estimated glomerular filtration rate [eGFR] < 60 mL/min/1.73 m^2^ based on at least two measurements taken 90 days apart, with or without markers of kidney damage [10]) and an NKF group (Figure 1A).

Patients diagnosed with acute hepatitis C, those with any liver disease other than CHC, those undergoing liver transplantation, those who had experienced drug interaction effects, particularly those with hepatitis B or human immunodeficiency virus, and those treated with other medications were excluded.

### 2.3. Outcomes Measured

DILI was the primary safety outcome of interest. Following Hy’s law for predicting serious hepatotoxicity, DILI adverse events were defined by the following criteria: [11,12] (i) Alanine aminotransferase (ALT) levels exceeding 5 times the upper limit of normal (ULN); (ii) Aspartate aminotransferase (AST) levels exceeding 5 times the ULN; (iii) ALT or AST levels greater than 3 times the ULN accompanied by total bilirubin (T-Bil) levels exceeding 2 times the ULN; (iv) ALT or AST levels greater than 3 times the ULN with an international normalized ratio (INR) above 1.5; (v) T-Bil levels exceeding 2 times the ULN with an INR above 1.5. Each case was followed until the first occurrence of any outcome measure or until the end of the study period. Cases where none of the specified outcomes occurred by the end of follow-up were classified as censored.

Additionally, the effectiveness of peritoneal dialysis was also assessed by analyzing the Sustained Virologic Response at 12 weeks (SVR12) within this patient cohort. Overall treatment effectiveness was determined by evaluating patients’ response to HCV therapy, with SVR12 defined as plasma HCV RNA levels falling below the lower limit of quantification (LLOQ) or less than 15 IU/mL, as measured by a quantitative test 12 weeks after completing treatment [13].

### 2.4. Sensitivity Analyses

We conducted two sensitivity analyses to further validate our findings. First, we redefined liver injury to encompass moderate to severe cases, based on the following criteria [14,15]: (a) ALT or AST levels at least three times the ULN; (b) Alkaline phosphatase (ALP) levels at least twice the ULN; (c) T-Bil levels at least twice the ULN in conjunction with elevated ALT or ALP levels; or (d) T-Bil levels at least twice the ULN with an INR of ≥1.5.

Second, to assess the potential impact of confounding by indication, we employed a case–time–control design [16]. In this design, the exposure assessment period was defined as the timeframe following the intervention index date, while the reference period was defined as the period preceding treatment. The study timeline was segmented into twenty-four distinct periods, determined by index dates, as laboratory test results were considered based on multiples of 7 days corresponding to follow-up visits. Treatment periods were categorized into pre-treatment (weeks 1–12) and post-treatment (weeks 1–12) phases (Figure 1B).

### 2.5. Statistical Analyses

Statistical analyses were conducted to assess the relationships and differences among study variables, ensuring methodological rigor in line with biomedical research standards. Categorical variables were summarized as frequencies and percentages, and their associations were evaluated using chi-square tests. Fisher’s exact test was applied in smaller sample sizes. This approach allowed for reliable inference, particularly in the context of categorical data with uneven distributions.

Continuous normal variables were expressed as mean ± standard deviation (SD) and compared by t-tests or ANOVA for repeated measures. Were performed using the Mann–Whitney U test or Kruskal–Wallis test (more robust under the violation of the normality assumption) for non-normally distributed variables [17,18].

The main outcome measure—hepatitis drug-induced liver injury (DILI)—was an-alyzed using a Poisson regression model, appropriate for count data and infrequent events. Relative risks were estimated as incidence rate ratios (IRRs), and adjustment for confounders was performed using logistic regression models. Adjusted Odds Ratios (aORs) were presented to describe the relationships between variables in a comprehensive manner [19,20].

Data missing were handled by applying multiple imputations based on mean-existing data, ensuring minimum bias and high statistical power [21]. Sensitivity analyses were performed to ensure the robustness of results under different assumptions and thresholds. Second, a case–time–control design was utilized as a sensitivity analysis to assess ORs across observation windows, attempting to address confounding by indication and temporal changes.

*p* < 0.05 was considered statistically significant and 95% confidence intervals (CIs) reported the precision of estimates. Statistical analyses were performed using SAS software version 9.4 (SAS Institute, Cary, NC, USA). These methods were chosen as they represent a balance between analytical rigor and the complexity that often accompanies clinical datasets in practice.

## 3. Results

### 3.1. Study Screening Process

**Figure 2:**
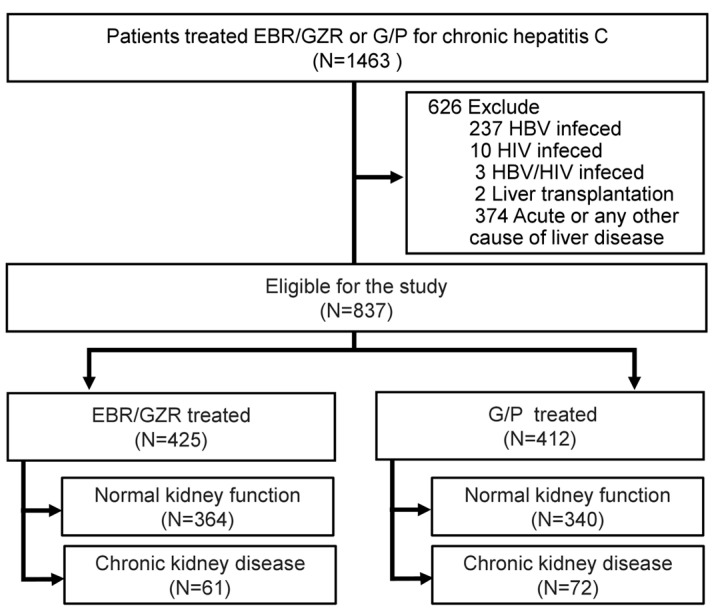
Flowchart of study population selection.

As shown in Figure 2, the study initially assessed 1463 patients who were treated with EBR/GZR or G/P for chronic hepatitis C between 1 August 2017 and 31 December 2020. Of these, 626 patients were excluded: 237 due to HBV co-infection, 10 due to HIV infection, 3 due to both HBV and HIV co-infection, 2 due to liver transplantation, and 374 due to acute or other causes of liver disease. The final cohort comprised 837 patients who met the inclusion criteria.

### 3.2. Baseline Characteristics

Table 1 summarizes the baseline characteristics of the study population, divided into patients with NKF (*n* = 735) and those with CKD (*n* = 102). The CKD group had a mean age of 65.85 years (SD = 10.00), compared to 59.09 years (SD = 13.36) in the NKF group, though this difference was not statistically significant (*p* = 0.683). Gender distribution was comparable between the two groups, with 43.27% males in the NKF group and 42.16% in the CKD group. A significant difference was noted in HCV genotype distribution, with genotype 1b being more prevalent in the CKD group (72.55% vs. 38.23%, *p* < 0.0001). No significant differences were observed in liver function markers (ALT, AST, T-Bil, INR) between the groups. However, the CKD group had a higher, although not statistically significant, incidence of cirrhosis (26.47% vs. 22.99%) and hepatic fibrosis (12.75% vs. 10.88%). Hyperlipidemia was significantly more common in the CKD group (22.55% vs. 18.23%, *p* = 0.042). Treatment duration of 12 weeks was similar between the groups (73.53% CKD vs. 72.93% NKF), as were the use of interferon (1.96% CKD vs. 1.22% NKF) and ribavirin (26.47% CKD vs. 27.03% NKF). Among CKD patients, the majority were classified as stage 3a or 3b, indicating moderate renal impairment.

Supplementary Table S1 provides further stratification of baseline characteristics by both renal function status and treatment regimen. Within the NKF group, there were no significant differences in age (G/P: 56.63 years, EBR/GZR: 61.85 years, *p* = 0.019) or gender distribution (G/P: 42.86% male, EBR/GZR: 43.64% male, *p* = 0.833) between those treated with G/P (*n* = 350) and those treated with EBR/GZR (*n* = 385). However, HCV genotype distribution differed significantly (*p* < 0.0001), with genotype 1b more common in the EBR/GZR group (47.80%) than in the G/P group (29.71%). Liver function markers and comorbidities, including hypertension (EBR/GZR: 42.60%, G/P: 42.29%, *p* = 0.934) and type 2 diabetes mellitus (T2DM) (EBR/GZR: 13.77%, G/P: 12.86%, *p* = 0.724), were similar across treatment groups.

In the CKD group, patients treated with G/P (*n* = 62) were slightly younger (63.22 years vs. 66.15 years, *p* = 0.014) than those treated with EBR/GZR (*n* = 40), though this difference was not statistically significant. The incidence of hepatic fibrosis (G/P: 12.90%, EBR/GZR: 10.00%) and cirrhosis (G/P: 29.03%, EBR/GZR: 25.00%) was higher in the CKD group compared to the NKF group, particularly in those treated with G/P, but these differences were not statistically significant. Hyperlipidemia was more frequently observed in the CKD group treated with G/P (22.58% vs. 10.00%, *p* = 0.019), while liver tumor incidence was higher in the CKD group treated with EBR/GZR (15.00% vs. 3.23%, *p* = 0.003). The diagnosis of comorbidities was based on diagnostic codes, as detailed in Supplementary Table S2.

### 3.3. SVR12 Outcomes in Peritoneal Dialysis Patients

**Figure 3:**
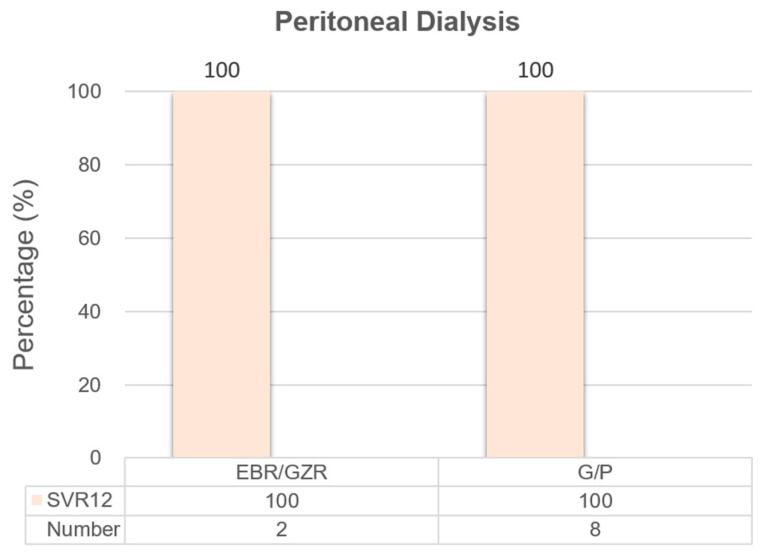
The effectiveness of peritoneal dialysis.

Figure 3 illustrates the SVR12 rates among patients undergoing peritoneal dialysis who were treated with either EBR/GZR or G/P. The SVR12 rate was 100% in both groups, with all patients (two in the EBR/GZR group and eight in the G/P group) achieving undetectable HCV RNA levels 12 weeks after completing therapy. These results suggest that both treatment regimens were highly effective in this patient population, despite the small sample size.

### 3.4. DILI Adverse Events

According to the standard criteria, 22 patients (2.63%) overall experienced a DILI event. When stratified by renal function, 19 patients (2.59%) in the NKF group and three patients (2.94%) in the CKD group met the criteria for DILI, though the difference was not statistically significant (*p* = 0.774). The most common event was ALT > 3 × ULN, which occurred in 12 patients (1.43%) overall, with 11 patients (1.50%) in the NKF group and one patient (0.98%) in the CKD group (*p* = 0.619). Additionally, ALT/AST > 3 × ULN with T-Bil > 2 × ULN was observed in two cases (0.24%) overall, with both cases occurring in the NKF group. The IRR for DILI events when comparing CKD to NKF patients under the standard criteria was 1.14 (95% CI: 0.34–3.84, *p* = 0.84), indicating no significant difference in the risk of DILI between the two groups (Table 2).

**Figure 4:**
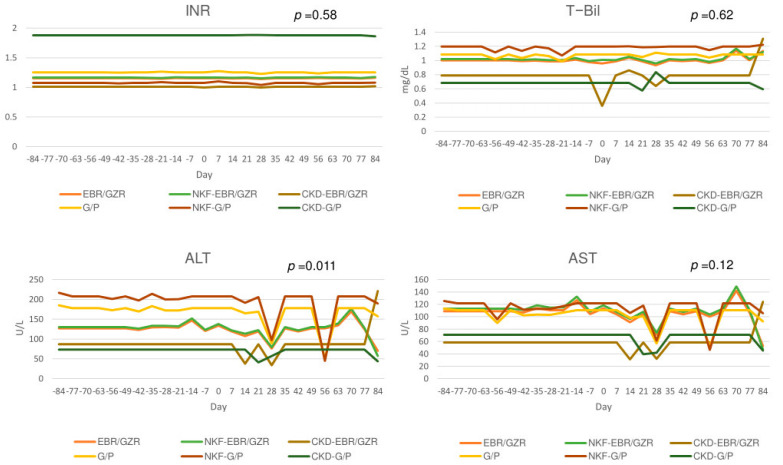
Laboratory parameters between pre-treatment and post-treatment in DILI event patients.

During the 12 weeks of medication, alterations were seen in patients’ ALT, AST, T-Bil levels, and INRs. There were significant differences in ALT, AST, T-Bil levels (*p* < 0.0001), and INR (*p* < 0.00071) between those with different severity of renal function impairment (Figure S1). At post-treatment weeks four and eight, ALT and AST levels plummeted in the NKF patients; whereas these levels started to decline from week 2 onward in patients with CKD. Interestingly, the levels were seen to gradually rise following their descent, especially in the EBR/GZR group, where peak levels were seen at week 10, followed by a slow decline. Only the ALT values were significantly different between treatment groups (*p* = 0.011) (Figure 4).

### 3.5. Sensitivity Analyses Outcomes

The sensitivity analysis revealed a higher incidence of DILI events, with 46 patients (5.50%) overall meeting the sensitivity criteria. Within the NKF group, 44 patients (5.99%) experienced DILI events, compared to two patients (1.96%) in the CKD group, although the difference did not reach statistical significance (*p* = 0.065). The most frequent event in the sensitivity analysis was ALT > 3 × ULN, reported in 29 patients (3.47%) overall, with 28 patients (3.81%) in the NKF group and 1 patient (0.98%) in the CKD group (*p* = 0.140). The IRR for DILI events in the sensitivity analysis was 3.05 (95% CI: 0.74–12.59, *p* = 0.12), suggesting a non-significant trend toward a higher risk of DILI in the CKD group under these criteria (Table 2).

We also assessed the odds ratios (ORs) for pre-treatment, post-treatment, and case–time–control to clarify indication confounding. The G/P group had a lower OR than EBR/GZR, both before and after intervention and regardless of liver damage severity; although the between-treatment differences were not significant (Table S3). Our case–time–control analyses enabled the rejection of the hypothesis that liver damage in HCV is caused by the medication. We found that patients on both EBR/GZR and G/P drugs had a lower risk of DILI events than patients during the non-treatment periods, with a higher risk during the pre-treatment than the post-treatment period. The higher risks during the pre-treatment periods, compared to the post-treatment period, in view of the patient in liver damage status.

### 3.6. Subgroup Analyses Outcomes

Detailed subgroup analyses were carried out to assess the incidence of DILI, the effect on ALT/AST levels, and treatment outcomes stratified by the following patient characteristics: renal function status, age, BMI, and the presence of comorbidities.

The incidence of DILI among patients with NKF and those with chronic CKD showed that 22 patients (2.63%) overall experienced DILI, with the EBR/GZR group showing a slightly higher incidence (3.06%) compared to the G/P group (2.18%). Within the NKF subgroup, DILI was observed in 19 patients (2.59%), with the highest incidence in the EBR/GZR group (3.12%). In the CKD subgroup, three patients (2.94%) developed DILI, with a marginally higher incidence in those treated with EBR/GZR (3.23%). There were no statistically significant differences between treatment groups in the IRRs across renal function subgroups despite these findings (Table S4). These results highlight that while both regimens are generally safe, subtle differences in DILI incidence warrant consideration in high-risk populations.

The sensitivity analysis focusing on ALT/AST level alterations revealed a higher overall incidence of ALT/AST elevations, with 47 patients (5.62%) affected. The incidence was slightly higher in the EBR/GZR group (6.23%) compared to the G/P group (5.58%). In the NKF subgroup, 44 individuals (5.99%) showed elevated ALT/AST levels, with the highest incidence observed in the EBR/GZR group (6.23%). Among CKD patients, three individuals (2.94%) exhibited ALT/AST elevations, primarily in the G/P group (3.23%). The IRRs for these events indicated no significant differences between the treatment regimens across all renal function subgroups (Table S5). These findings underscore the importance of monitoring liver function closely during therapy, especially in patients at high risk of liver enzyme abnormalities, for early identification and effective management.

Further analysis of the 22 patients who developed DILI revealed that the majority of cases occurred in older patients, particularly those over 65 years, and in those with advanced CKD (Stage 3b and Stage 4). Notably, SVR was 100% in both the G/P and EBR/GZR groups among patients who experienced DILI (Table S6).

The risk of DILI in relation to comorbidities and co-administered drugs showed that significant risk factors included liver tumors (Adjusted OR: 18.89, *p* < 0.001) and hypertension (Adjusted OR: 4.25, *p* = 0.007) (Table S7). These findings highlight the significance of utilizing patient-specific risk factors in the initiation of treatment, particularly in patients with multiple comorbidities. Importantly, all patients who had Adverse Liver Events (DILI) developed SVR indicating that liver events did not attenuate antiviral efficacy.

Furthermore, we examined the treatment effectiveness of patients other than the population of peritoneal dialysis according to baseline characteristics, including gender, HCV genotype, CKD stage, age, BMI, and comorbidities.

Treatment effectiveness in the intention-to-treat (ITT) population indicated an overall SVR rate of 91.78%, with higher success in the G/P group (99.27%) compared to EBR/GZR (86.35%, *p* < 0.0001). The loss to follow-up rate was significantly higher in the EBR/GZR group, especially among CKD patients (11.77% vs. 1.61%, *p* = 0.005), suggesting improved tolerability or adherence to G/P therapy (Table S8). The G/P regimen consistently outperformed EBR/GZR, especially for older patients, those with advanced CKD, or significant comorbidities (e.g., liver tumors, hypertension). Outperformance here reinforces the G/P regimen’s broad versatility and effectiveness for complex clinical needs (Table S9).

In summary, these subgroup analyses reveal that both G/P and EBR/GZR are effective treatments, with G/P showing a trend toward better outcomes in specific subgroups, including patients with more advanced CKD, older age, and certain comorbidities. The analyses also highlight significant risk factors for DILI, emphasizing the need for careful consideration of patient characteristics when selecting treatment regimens.

## 4. Discussion

In this study, we demonstrated that both G/P and EBR/GZR regimens are highly effectiveness in treating peritoneal dialysis groups. Importantly, no significant differences in the IRRs of the DILI were observed between patients with or without CKD across both treatment groups, even when sensitivity analyses with altered hepatic biochemical standards were applied. This finding underscores the safety profile of these therapies, even in populations with advanced renal dysfunction. However, additional sensitivity analyses revealed that comorbid conditions, specifically liver tumors, and hypertension, were significantly associated with an elevated risk of DILI, underscoring the importance of considering these factors in patient management. While there was a minor correlation between the occurrence of DILI and DAA treatment, the presence of these comorbidities likely acted as significant con-founding factors.

One of the key strengths of our study is the use of real-world data, which provides a practical perspective on the effectiveness and safety of DAAs in patients with CKD, including those undergoing peritoneal dialysis. Additionally, our detailed subgroup analyses and the inclusion of two sensitivity analyses allow for a nuanced understanding of treatment outcomes across various patient profiles and help validate the robustness of our findings. However, our study has limitations, including incomplete data linkage, which may have affected follow-up accuracy, and potential selection bias due to the exclusion of patients with severe liver impairment.

Our study identified hypertension and liver tumors as significant risk factors for DILI during treatment with DAAs. These findings corroborate earlier research that highlighted the role of comorbidities in influencing liver function during antiviral therapy [22]. Clinicians should prioritize early identification of such risk factors and employ proactive monitoring strategies to mitigate potential adverse effects.

Some evidence aligns with our findings that DILI events are significantly influenced by pre-existing risk factors related to the underlying illness. Within our study cohort, no significant deterioration in liver function was observed during the treatment period. However, this result may be influenced by selection bias, as current guidelines for DAA therapy generally exclude patients with severe liver impairment. A real-world study from Taiwan reported elevated ALT levels in 10.9% of HCV patients receiving DAAs, with the lowest incidence found in those treated with G/P at 5.4%. Notably, HBV co-infection and elevated pre-treatment ALT levels were identified as significant predictors of increased ALT during treatment [22]. Furthermore, a retrospective cohort study focusing on G/P in chronic hepatitis C patients found that the frequency of G/P-induced grade 3 or higher liver injury, measured by T-Bil, AST, ALT, ALP, and GGT, was 3.8%, 0%, 0%, 0%, and 0.4%, respectively [23]. Moreover, data from the FDA Adverse Event Reporting System included 63 reports of DILI, where some patients had mild liver impairment (Child–Pugh A) at baseline. However, over half of these cases were likely misclassified, as they involved patients with substantial pre-existing risk factors, such as hepatic carcinoma or other severe liver-related conditions. These pre-existing factors may have contributed to the observed clinical deterioration in liver function or liver failure during the administration of HCV therapies [24]. Furthermore, in a previous study, seven peritoneal dialysis patients treated with G/P for an average of 11.2±1.8 weeks experienced favorable outcomes, although one patient died of PD-related peritonitis during treatment [25].

Hypertension emerged as a risk factor for DILI in our study, though the underlying reasons for this association are not entirely clear from the existing literature. Hypertension is a common comorbidity, and a Japanese study has indicated that patients with CHC tend to have a higher prevalence of comorbid conditions and are prescribed more medications than those without HCV, which may contribute to the elevated risk. In CHC patients, the most frequent comorbidities include gastrointestinal diseases, hypertensive disorders, and metabolic abnormalities. Additionally, a U.S. study reported an essential hypertension prevalence of 32.6% among CHC patients [26].

When evaluating DILI events related to other DAA regimens, our previous retrospective analysis found that the risk of DILI with EBR/GZR was 2.84 times higher (95% CI: 0.71–11.35) compared to ombitasvir/paritaprevir/ritonavir/dasabuvir, though this difference was not statistically significant. Notably, the addition of RBV to these regimens did not lead to an increased incidence of DILI [27]. Additionally, given that genotype 1b is the most prevalent strain of HCV in Taiwan, the study’s findings that highlight its significance in the region are particularly relevant [28].

There were several limitations to our study. First, we were unable to link the de-identified cohort with death or cancer registries in the DRD, resulting in incomplete information for patients lost to follow-up. Second, as the Taiwanese government expanded the criteria for treating HCV patients and began covering the costs of DAA treatment, the volume of data we collected increased, but it became less detailed and incomplete. For example, we did not have access to comprehensive baseline information regarding the severity of hepatic disease and the presence of advanced liver disease. Third, in Taiwan, EBR/GZR and G/P are contraindicated for patients with moderate or severe hepatic insufficiency (Child–Pugh B or C), which may have introduced selection bias by excluding patients with cirrhosis. Finally, we lacked access to certain data, such as hemodialysis records and laboratory test results, including hemoglobin, γ-glutamyl transpeptidase, α-fetoprotein, and alkaline phosphatase levels. As a result, we could not utilize ALP and ALT levels to calculate R-values and further assess the types of liver injury.

## 5. Conclusions

This study highlights the effectiveness and safety of G/P and EBR/GZR as novel therapies for hepatitis C treatment in patients with peritoneal dialysis. The results show that DAA therapy is not associated with treatment-related hepatotoxicity and that the main causes of abnormal liver parameters achieved during the DAA therapy are pre-existing hepatic-related conditions, mainly associated with the other comorbidities and not the antiviral agents themselves. This highlights the necessity for comprehensive pre-treatment evaluations and individualized treatment strategies, particularly for high-risk patients.

Furthermore, despite being rare, liver damage during DAA therapy should nontheless prompt close monitoring of liver parameters during treatment and the reason in the setting of complications emerging. Examples of preventive measures and vigilant surveillance that can be put into practice to help ensure patient safety and treatment outcomes. Future studies should assess long-term outcomes, pharmacokinetic adjustments for dialysis-dependent patients, and predictive biomarkers of adverse events. Furthermore, relative comparison of DAAs with novel therapeutic agents will be essential to further treatment paradigms in this area. These research avenues are intended to optimize care for individuals with CHC and complex comorbidities.

## Figures and Tables

**Table 1:** Patient characteristics.

	Normal Kidney Function (*n* = 735)		Chronic Kidney Disease (*n* = 102)		*p* -Value
Age, mean (SD)	59.09	(13.36)	65.65	(10.00)	0.69
BMI, kg/m^2^, mean (SD)	24.17	(4.21)	24.43	(5.06)	0.23
Gender, *n* (%)					0.64
Male	318	(43.27)	43	(42.16)	0.20
Female	417	(56.73)	59	(57.84)	0.04 *
HCV genotype, *n* (%)					
1a	9	(1.23)	1	(0.98)	0.42
1b	289	(39.37)	37	(36.27)	<0.001 *
1+2	2	(0.27)	0	(0.00)	-
Undetected	437	(59.46)	64	(62.75)	<0.001 *
HCV RNA, log10IU/mL mean (SD)	6.55	(6.67)	6.34	(6.51)	0.99
ALT, IU/L (SD)	66.67	(73.40)	38.80	(37.77)	<0.001 *
AST, IU/L (SD)	55.40	(49.74)	37.42	(25.67)	<0.001 *
T-Bil, mg/dL (SD)	0.70	(0.32)	0.40	(0.21)	<0.001 *
INR (SD)	1.04	(0.09)	1.05	(0.23)	0.76
Therapy duration at weeks, *n* (%)					
8	536	(72.93)	75	(73.53)	<0.001 *
12	199	(27.07)	27	(26.47)	<0.001 *
IFN	0	(0.00)	0	(0.00)	-
RBV combined	0	(0.00)	0	(0.00)	-
Cirrhosis, *n* (%)	15	(2.04)	0	(0.00)	-
Hepatic fibrosis, *n* (%)	66	(8.98)	19	(18.63)	0.45
Peritoneal Dialysis	0	(0.00)	10	(9.80)	0.19
Renal function, *n* (%)	735	(87.81)	102	(12.19)	0.57
Stage1	543	(73.88)	0	(0.00)	-
Stage 2 (mild)	192	(26.12)	0	(0.00)	-
Stage 3a (moderate)	0	(0.00)	24	(23.53)	-
Stage 3b (moderate)	0	(0.00)	12	(11.76)	-
Stage 4 (severe)	0	(0.00)	12	(11.76)	-
Stage 5	0	(0.00)	54	(52.94)	-
Comorbidities, *n* (%)					
Liver tumor	40	(5.44)	8	(7.84)	0.92
Digestive system neoplasms	113	(15.37)	15	(14.71)	0.28
T2DM	16	(2.18)	1	(0.98)	0.21
Hyperlipidaemia	89	(12.11)	12	(11.76)	0.28
Hypertension	159	(21.63)	58	(56.86)	0.76
Peptic ulcer	252	(34.29)	39	(38.24)	0.01 *
Gastric ulcer	222	(30.20)	25	(24.51)	0.93
Constipation	118	(16.05)	26	(25.49)	0.71
Dizziness and giddiness	119	(16.19)	23	(22.55)	0.33
Functional dyspepsia	108	(14.69)	12	(11.76)	0.42
GERD with esophagitis	115	(15.65)	18	(17.65)	0.03 *
Acute abdomen	113	(15.37)	15	(14.71)	0.61
UTI	103	(14.01)	20	(19.61)	0.56
Sleep disorder	77	(10.48)	14	(13.73)	0.04 *
Anxiety disorder	69	(9.39)	9	(8.82)	0.27
Peptic ulcer with hemorrhage	52	(7.07)	7	(6.86)	0.16
Irritable bowel syndrome	129	(17.55)	14	(13.73)	0.77
Flatulence	34	(4.63)	9	(8.82)	0.71
Medication 12 months before starting treatment, *n* (%)					
Silymarin	276	(37.55)	40	(39.22)	0.48
Acetaminophen 500 mg	164	(22.31)	41	(40.20)	0.39
Famotidine 20 mg	190	(25.85)	41	(40.20)	0.66
Fursultiamine (TTFD) 50 mg, Riboflavin 5 mg	154	(20.95)	23	(22.55)	0.62
Proheparum tab	60	(8.16)	6	(5.88)	0.24
Pantoprazole 40 mg	115	(15.65)	15	(14.71)	0.61
Dimethylpolysiloxane 40 mg	115	(15.65)	14	(13.73)	0.37
Mosapride citrate 5 mg	98	(13.33)	12	(11.76)	0.41
Sennosides 20 mg	75	(10.20)	29	(28.43)	0.78

EBR/GZR, elbasvir/grazoprevir; G/P, glecaprevir/pibrentasvir; SD, standard deviation; BMI, Body Mass Index; ALT, AL-anine aminotransferase; AST, aspartate aminotransferase; TB, INR, total bilirubin; international normalized ratio; IFN, interferon; RBV, ribavirin; CKD, Chronic kidney disease; T2DM, Type 2 diabetes mellitus; GERD, Gastroesophageal reflux disease; UTI, Urinary Tract Infection. * Significant difference (*p* < 0.05).

**Table 2:** Incidence of drug-induced liver injury adverse events.

	**Overall**		**NKF**		**CKD**		***p*** **-Value**
	***n***	**(%)**	***n***	**(%)**	***n***	**(%)**	
Standards criteria of DILI							
Event	22	(2.63)	19	(2.59)	3	(2.94)	0.74
ALT > 5 × ULN	12	(1.43)	11	(1.5)	1	(0.98)	0.68
AST > 5 × ULN	7	(0.84)	6	(0.82)	1	(0.98)	0.6
ALT/AST > 3 × ULN +T-Bil > 2 × ULN †	2	(0.24)	2	(0.27)	0	(0)	0.6
ALT/AST > 3 × ULN + INR > 1.5	2	(0.24)	1	(0.14)	1	(0.98)	0.23
T-Bil > 2 × ULN + INR > 1.5	2	(0.24)	2	(0.27)	0	(0)	0.6
Sensitivity analysis							
Event	46	(5.5)	44	(5.99)	2	(1.96)	0.11
ALT > 3 × ULN	30	(3.58)	28	(3.81)	2	(1.96)	0.57
AST > 3 × ULN	36	(4.3)	34	(4.63)	2	(1.96)	0.3
ALT/AST > 3 × ULN + T-Bil > 2 × ULN †	2	(0.24)	2	(0.27)	0	(0)	0.6
ALT/AST > 3 × ULN + INR > 1.5	1	(0.12)	1	(0.14)	0	(0)	0.71
T-Bil > 2 × ULN + INR > 1.5	2	(0.24)	2	(0.27)	0	(0)	0.6
	**IRR**			**(95% CI)**			***p*** **-Value**
Standards criteria of DILI							
Event	1.14			(0.34–3.84)			0.84
ALT > 5 × ULN	1.53			(0.2–11.82)			0.69
AST > 5 × ULN	0.83			(0.1–6.92)			0.87
ALT/AST > 3 × ULN +T-Bil > 2 × ULN †	-			-			-
ALT/AST > 3 × ULN + INR > 1.5	-			-			-
T-Bil > 2 × ULN + INR > 1.5	0.14			(0.01–2.22)			0.16
Sensitivity analysis							
Event	3.05			(0.74–12.59)			0.12
ALT > 3 × ULN	1.94			(0.46–8.16)			0.36
AST > 3 × ULN	2.36			(0.57–9.82)			0.24
ALT/AST > 3 × ULN +T-Bil > 2 × ULN †	-			-			-
ALT/AST > 3 × ULN + INR > 1.5	-			-			-
T-Bil > 2 × ULN + INR > 1.5	-			-			-

EBR/GZR, elbasvir/grazoprevir; G/P, glecaprevir/pibrentasvir; ALT, alanine aminotransferase; AST, aspartate aminotransferase; T-Bil, total bilirubin; INR, international normalized ratio; IFN, interferon; RBV, ribavirin; IRR, incidence rate ratio; CI, confidence interval; ULN, upper limit of normal. † Including the patients with ALT > 5 times ULN.

## Data Availability

The original contributions presented in this study are included in the article/Supplementary Material. Further inquiries can be directed to the corresponding author.

## Supplementary Materials

The following supporting information can be downloaded at: https://www.mdpi.com/article/10.3390/biomedicines13010055/s1. Table S1: Patient characteristics in overall; Table S2: Risk of DILI event in different study periods; Table S3: The patients of DILI event characteristics; Table S4: Evaluate the DILI event risk of comorbidities and co-administered drug; Table S5: Population in effectiveness analysis (ITT); Table S6: Successful cure rate for different categories at baseline (ITT); Table S7: Evaluate the DILI event risk of comorbidities and co-administered drug; Table S8: Population in effectiveness analysis (ITT); Table S9: Successful cure rate for different categories at baseline (ITT); Figure S1: Laboratory assessments.
