# Supplementary material for: Direct-Acting Antivirals in Hepatitis C Treatment for Renal Impairment: Liver Safety Concerns and Effectiveness in Peritoneal Dialysis
Source: Biomedicines. 2024 Dec 29;13(1):55. doi: 10.3390/biomedicines13010055 (PMC11760478; doi:10.3390/biomedicines13010055)
Supplement: Supplementary file 1 [file biomedicines-13-00055-s001.zip › biomedicines-3394181-supplementary.pdf]

**Direct-Acting Antivirals in Hepatitis C Treatment for Renal Impairment: Liver Safety Concerns and Effectiveness in Peri-toneal Dialysis**

Hsuan-Yu Hung [1,2], Wei-Liang Hung [3], Ye Gu [4] and Chung-Yu Chen[5,6,7]

[1]School of Pharmacy, College of Pharmacy, Kaohsiung Medical University, Kaohsiung, Taiwan; [2]Department of Pharmacy, Ditmanson Medical Foundation Chia-Yi Christian Hospital, Chiayi, Taiwan; [3]Division of Nephrology, Department of Medicine, Zuoying Armed Forces General Hospital, Kaohsiung, Taiwan;[4]Graduate Institute of Natural Products, College of Pharmacy, Kaohsiung Medical University, Kaohsiung, Taiwan; [5] Master Program in Clinical Pharmacy, School of Pharmacy, Kaohsiung Medical University, Kaohsiung, Taiwan; [6] Department of Medical Research, Kaohsiung Medical University Hospital, Kaohsiung, Taiwan; [7] Department of Pharmacy, Kaohsiung Medical University Hospital, Kaohsiung, Taiwan.

Corresponding author: Chung-Yu Chen (jk2975525@hotmail.com)

**Contents**

Table S1. Baseline characteristics of patients stratified by renal function and treatment regimen ............. 2
Table S2. Determine the diagnosis for comorbidities ........................................................................ 4
Table S3. Risk of DILI event in different study periods ....................................................................... 5
Table S6. The patients of DILI event characteristics ........................................................................... 8
Table S7. Evaluate the DILI event risk of comorbidities and co-administered drug.................................. 10
Table S8. Population in effectiveness analysis (ITT). ......................................................................... 12
Table S9. Successful cure rate for different categories at baseline (ITT)................................................... 13
Figure S1. Laboratory assessments.......................................................................................................... 15
Reference ..........................................................................................................**Error! Bookmark not defined.**

**Table S1. Baseline characteristics of patients stratified by renal function and treatment regimen**

| | Normal kidney function | | | | | | | Chronic kidney disease | | | | | | |
|---|---|---|---|---|---|---|---|---|---|---|---|---|---|---|
| | Total (*n* = 735) | | G/P (*n* = 350) | | EBR/GZR (*n* = 385) | | *p - value* | Total (*n* = 102) | | G/P (*n* = 62) | | EBR/GZR (*n* = 40) | | *p - value* |
| Age, mean (SD) | 59.09 | (13.36) | 56.36 | (13.78) | 61.58 | (12.48) | <0.001* | 65.65 | (10.00) | 65.32 | (10.20) | 66.15 | (9.78) | 0.69 |
| BMI, kg/m$^2$, mean (SD) | 24.17 | (4.21) | 23.69 | (4.38) | 24.47 | (4.08) | 0.19 | 24.43 | (5.06) | 25.23 | (5.22) | 23.20 | (4.70) | 0.23 |
| Gender, *n* (%) | | | | | | | 0.83 | | | | | | | 0.64 |
| Male | 318 | (43.27) | 150 | (42.86) | 168 | (43.64) | | 43 | (42.16) | 55 | (40.32) | 18 | (45.00) | |
| Female | 417 | (56.73) | 200 | (57.14) | 217 | (56.36) | | 59 | (57.84) | 37 | (59.68) | 22 | (55.00) | |
| HCV genotype, *n* (%) | | | | | | | | | | | | | | |
| 1a | 9 | (1.23) | 8 | (2.29) | 1 | (0.26) | 0.01* | 1 | (0.98) | 1 | (1.61) | 0 | (0.00) | 0.42 |
| 1b | 289 | (39.37) | 67 | (19.20) | 222 | (57.66) | <0.001* | 37 | (36.27) | 11 | (17.74) | 26 | (65.00) | <0.001* |
| 1+2 | 2 | (0.27) | 2 | (0.57) | 0 | 0.00 | 0.14 | 0 | (0.00) | 0 | (0.00) | 0 | (0.00) | |
| Undetected | 437 | (59.46) | 275 | (78.57) | 162 | (42.08) | <0.001* | 64 | (62.75) | 50 | (80.65) | 14 | (35.00) | <0.001* |
| HCV RNA, log10IU/mL mean (SD) | 6.55 | (6.67) | 6.56 | (6.62) | 6.54 | (6.71) | 0.76 | 6.34 | (6.51) | 6.37 | (6.54) | 6.25 | (6.43) | 0.60 |
| ALT, IU/L (SD) | 66.67 | (73.40) | 68.76 | (89.46) | 64.81 | (55.31) | 0.01* | 38.80 | (37.77) | 36.45 | (35.10) | 42.54 | (41.91) | 0.13 |
| AST, IU/L (SD) | 55.40 | (49.74) | 53.06 | (52.62) | 57.47 | (47.02) | <0.001* | 37.42 | (25.67) | 36.34 | (23.89) | 39.14 | (28.53) | 0.32 |
| T-Bil, mg/dL (SD) | 0.70 | (0.32) | 0.67 | (0.29) | 0.73 | (0.34) | 0.01* | 0.40 | (0.21) | 0.41 | (0.22) | 0.39 | (0.18) | 0.92 |
| INR (SD) | 1.04 | (0.09) | 1.04 | (0.10) | 1.04 | (0.08) | 0.84 | 1.05 | (0.23) | 1.08 | (0.28) | 1.00 | (0.06) | 0.07 |
| Therapy duration at weeks, *n* (%) | | | | | | | | | | | | | | |
| 8 | 536 | (72.93) | 338 | (96.57) | 198 | (51.43) | <0.001* | 75 | (73.53) | 55 | (88.71) | 20 | (50.00) | <0.001* |
| 12 | 199 | (27.07) | 12 | (3.43) | 187 | (48.57) | <0.001* | 27 | (26.47) | 7 | (11.29) | 20 | (50.00) | <0.001* |
| IFN | 0 | (0.00) | 0 | (0.00) | 0 | (0.00) | - | 0 | (0.00) | 0 | (0.00) | 0 | (0.00) | - |
| RBV combined | 0 | (0.00) | 0 | (0.00) | 0 | (0.00) | - | 0 | (0.00) | 0 | (0.00) | 0 | (0.00) | - |
| Cirrhosis, *n* (%) | 15 | (2.04) | 5 | (1.43) | 10 | (2.60) | 0.26 | 0 | (0.00) | 0 | (0.00) | 0 | (0.00) | - |
| Hepatic fibrosis, *n* (%) | 66 | (8.98) | 19 | (5.43) | 47 | (12.21) | 0.01* | 19 | (18.63) | 13 | (20.97) | 6 | (15.00) | 0.45 |
| Peritoneal Dialysis | 0 | (0.00) | 0 | (0.00) | 0 | (0.00) | | 10 | (9.80) | 8 | (12.90) | 2 | (5.00) | 0.19 |
| Normal kidney function | 735 | (100.00) | 350 | (47.62) | 385 | (52.38) | 0.28 | | | | | | | |
| CKD | | | | | | | | 102 | (100.00) | 62 | (60.78) | 40 | (39.22) | 0.57 |
| Stage1 | 543 | (73.88) | 265 | (75.71) | 278 | (72.21) | - | 0 | (0.00) | 0 | (0.00) | 0 | (0.00) | - |
| Stage 2 (mild) | 192 | (26.12) | 85 | (24.29) | 107 | (27.79) | - | 0 | (0.00) | 0 | (0.00) | 0 | (0.00) | - |
| Stage 3a (moderate) | 0 | (0.00) | 0 | (0.00) | 0 | (0.00) | - | 24 | (23.53) | 14 | (22.58) | 10 | (25.00) | - |
| Stage 3b (moderate) | 0 | (0.00) | 0 | (0.00) | 0 | (0.00) | - | 12 | (11.76) | 6 | (9.68) | 6 | (15.00) | - |
| Stage 4 (severe) | 0 | (0.00) | 0 | (0.00) | 0 | (0.00) | - | 12 | (11.76) | 6 | (9.68) | 6 | (15.00) | - |
| Stage 5 | 0 | (0.00) | 0 | (0.00) | 0 | (0.00) | - | 54 | (52.94) | 36 | (58.06) | 18 | (45.00) | - |
| Comorbidities, *n* (%) | | | | | | | | | | | | | | |
| Liver tumor | 40 | (5.44) | 10 | (2.86) | 30 | (7.79) | 0.01* | 8 | (7.84) | 5 | (8.06) | 3 | (7.50) | 0.92 |

| | | | | | | | | | | | | | | |
|---|---|---|---|---|---|---|---|---|---|---|---|---|---|---|
| Digestive system neoplasms | 113 | (15.37) | 36 | (10.59) | 77 | (20.00) | <0.001* | 15 | (14.71) | 11 | (17.74) | 4 | (10.00) | 0.28 |
| T2DM | 16 | (2.18) | 3 | (0.86) | 13 | (3.38) | 0.02* | 1 | (0.98) | 0 | 0.00 | 1 | (2.50) | 0.21 |
| Hyperlipidaemia | 89 | (12.11) | 31 | (8.86) | 58 | (15.06) | 0.01* | 12 | (11.76) | 9 | (14.52) | 3 | (7.50) | 0.28 |
| Hypertension | 159 | (21.63) | 54 | (15.43) | 105 | (27.27) | <0.001* | 58 | (56.86) | 36 | (58.06) | 22 | (55.00) | 0.76 |
| Peptic ulcer | 252 | (34.29) | 88 | (25.14) | 164 | (42.60) | <0.001* | 39 | (38.24) | 30 | (48.39) | 9 | (22.50) | 0.01* |
| Gastric ulcer | 222 | (30.20) | 99 | (28.29) | 123 | (31.95) | 0.28 | 25 | (24.51) | 15 | (24.19) | 10 | (25.00) | 0.93 |
| Constipation | 118 | (16.05) | 43 | (12.29) | 75 | (19.48) | 0.01* | 26 | (25.49) | 15 | (24.19) | 11 | (27.50) | 0.71 |
| Dizziness and giddiness | 119 | (16.19) | 45 | (12.86) | 74 | (19.22) | 0.02* | 23 | (22.55) | 16 | (25.81) | 7 | (17.50) | 0.33 |
| Functional dyspepsia | 108 | (14.69) | 36 | (10.29) | 72 | (18.70) | 0.01* | 12 | (11.76) | 6 | (9.68) | 6 | (15.00) | 0.42 |
| GERD with esophagitis | 115 | (15.65) | 37 | (10.57) | 78 | (20.26) | <0.001* | 18 | (17.65) | 15 | (24.19) | 3 | (7.50) | 0.03* |
| Acute abdomen | 113 | (15.37) | 48 | (13.71) | 65 | (16.88) | 0.23 | 15 | (14.71) | 10 | (16.13) | 5 | (12.50) | 0.61 |
| UTI | 103 | (14.01) | 37 | (10.57) | 66 | (17.14) | 0.01* | 20 | (19.61) | 11 | (17.74) | 9 | (22.50) | 0.56 |
| Sleep disorder | 77 | (10.48) | 29 | (8.53) | 48 | (12.47) | 0.07 | 14 | (13.73) | 12 | (19.35) | 2 | (5.00) | 0.04* |
| Anxiety disorder | 69 | (9.39) | 26 | (7.43) | 43 | (11.17) | 0.08 | 9 | (8.82) | 7 | (11.29) | 2 | (5.00) | 0.27 |
| peptic ulcer with hemorrhage | 52 | (7.07) | 18 | (5.14) | 34 | (8.83) | 0.05 | 7 | (6.86) | 6 | (9.68) | 1 | (2.50) | 0.16 |
| Irritable bowel syndrome | 129 | (17.55) | 58 | (16.57) | 71 | (18.44) | 0.51 | 14 | (13.73) | 9 | (14.52) | 5 | (12.50) | 0.77 |
| Flatulence | 34 | (4.63) | 12 | (3.43) | 22 | (5.71) | 0.14 | 9 | (8.82) | 6 | (9.68) | 3 | (7.50) | 0.71 |
| Medication 12 months before starting treatment, *n* (%) | | | | | | | | | | | | | | |
| Silymarin | 276 | (37.55) | 99 | (28.29) | 177 | (45.97) | <0.001* | 40 | (39.22) | 26 | (41.94) | 14 | (35.00) | 0.48 |
| Acetaminophen 500mg | 164 | (22.31) | 68 | (19.43) | 96 | (24.94) | 0.07 | 41 | (40.20) | 27 | (43.55) | 14 | (35.00) | 0.39 |
| Famotidine 20 mg | 190 | (25.85) | 69 | (19.71) | 121 | (31.43) | <0.001* | 41 | (40.20) | 26 | (41.94) | 15 | (37.50) | 0.66 |
| Fursultiamine (TTFD) 50mg, Riboflavin 5mg | 154 | (20.95) | 66 | (18.86) | 88 | (22.86) | 0.18 | 23 | (22.55) | 15 | (24.19) | 8 | (20.00) | 0.62 |
| Proheparum tab | 60 | (8.16) | 19 | (5.43) | 41 | (10.65) | 0.01* | 6 | (5.88) | 5 | (8.06) | 1 | (2.50) | 0.24 |
| Pantoprazole 40 mg | 115 | (15.65) | 37 | (10.57) | 78 | (20.26) | <0.001* | 15 | (14.71) | 10 | (16.13) | 5 | (12.50) | 0.61 |
| Dimethylpolysiloxane 40mg | 115 | (15.65) | 54 | (15.43) | 61 | (15.84) | 0.88 | 14 | (13.73) | 7 | (11.29) | 7 | (17.50) | 0.37 |
| Mosapride citrate 5 mg | 98 | (13.33) | 35 | (10.00) | 63 | (16.36) | 0.01* | 12 | (11.76) | 6 | (9.68) | 6 | (15.00) | 0.41 |
| Sennosides 20 mg | 75 | (10.20) | 21 | (6.00) | 54 | (14.03) | <0.001* | 29 | (28.43) | 17 | (27.42) | 12 | (30.00) | 0.78 |

EBR/GZR, elbasvir/grazoprevir; G/P, glecaprevir / pibrentasvir; SD, standard deviation; BMI, Body Mass Index; ALT, ALanine aminotransferase; AST, aspartate aminotransferase; TB, INR, total bilirubin; international normalized ratio; IFN, interferon; RBV, ribavirin; CKD, Chronic kidney disease; T2DM, Type 2 diabetes mellitus; GERD, Gastroesophageal reflux disease; UTI, Urinary Tract Infection.
*Significant difference ($p < 0.05$).

**Table S2. Determine the diagnosis for comorbidities**

| Comorbidities | ICD-9-CM | ICD-10-CM |
|---|---|---|
| Chronic kidney disease | 585 586 593.9 | N18.1 N18.2 N18.3 N18.4 N18.5 N18.6 N18.9 N19 |
| Liver tumor | 155 155.1 | C22.0 C22.2 C22.3 C22.4 C22.7 C22.8 C22.1 |
| Cirrhosis | 571.5 571.6 571.8 571.9 571.2 | K74.0 K74.1 K74.2 K74.60 K74.69 K74.3 K74.4 K74.5 K76.0 K70.30 K70.31 K70.32 K70.38 K70.39 |
| Hepatic fibrosis | 571.5 | K74.00 K74.01 K74.02 |
| Digestive system neoplasms | 239 235.2 235.3 235.5 | D49.0 D37.1 D37.2 D37.3 D37.4 D37.5 D37.6 D37.8 D37.9 |
| Type 2 diabetes mellitus | 250.00 250.02 250.10 250.12 250.20 250.22 250.30 250.32 250.40 250.42 250.50 250.52 250.60 250.62 250.70 250.72 250.80 250.82 250.90 250.92 357.2 362.01 362.02 366.41 583.81 | E11.00 E11.01 E11.21 E11.22 E11.29 E11.311 E11.319 E11.321 E11.329 E11.331 E11.339 E11.341 E11.349 E11.351 E11.359 E11.36 E11.39 E11.40 E11.41 E11.42 E11.43 E11.44 E11.49 E11.51 E11.52 E11.59 E11.610 E11.618 E11.620 E11.621 E11.622 E11.628 E11.630 E11.638 E11.641 E11.649 E11.65 E11.69 E11.8 E11.9 |
| Hyperlipidaemia | 272 272.1 272.2 272.3 272.4 272.5 272.8 272.9 759.89 | E78.0 E78.1 E78.2 E78.3 E78.4 E78.5 E78.6 E78.79 E78.89 E78.70 E78.9 E78.71 E78.72 |
| Hypertension | 401 401.1 401.9 402 402.1 402.9 402.01 402.11 402.91 403.01 403.11 403.91 403 403.1 403.9 404.01 404.11 404.91 404 404.1 404.9 404.02 404.12 404.92 404.03 404.13 404.93 405.01 405.11 405.91 405.99 405.09 405.19 | I10 I11.9 I11.0 I12.0 I12.9 I13.0 I13.10 I13.11 I13.2 I15.0 I15.1 I15.2 I15.8 I15.9 I16.0 I16.1 I16.9 |

**Table S3. Risk of DILI event in different study periods**

| | Pre-treatment | | Post-treatment | | Case-time-control | |
|---|---|---|---|---|---|---|
| | OR | (95% CI) | OR | (95% CI) | OR | (SE) |
| Overall | | | | | | |
| 1 week | 1.24 | (0.38 - 4.10) | 0.62 | (0.15 - 2.60) | 0.06 | (0.00033) |
| 2 weeks | 0.75 | (0.30 - 1.87) | 0.62 | (0.15 - 2.60) | 0.78 | (0.00013) |
| 3 weeks | 0.89 | (0.42 - 1.90) | 0.62 | (0.15 - 2.60) | 0.59 | (0.00011) |
| 4 weeks | 0.89 | (0.42 - 1.90) | 1.03 | (0.30 - 3.59) | 0.59 | (0.00011) |
| 5 weeks | 1.10 | (0.55 - 2.21) | 1.03 | (0.30 - 3.59) | 0.52 | (0.00011) |
| 6 weeks | 1.28 | (0.67 - 2.42) | 1.03 | (0.33 - 3.23) | 0.50 | (0.00011) |
| 7 weeks | 1.28 | (0.67 - 2.42) | 1.03 | (0.36 - 2.97) | 0.50 | (0.00011) |
| 8 weeks | 1.34 | (0.71 - 2.52) | 1.03 | (0.38 - 2.78) | 0.50 | (0.00011) |
| 9 weeks | 1.40 | (0.75 - 2.62) | 0.93 | (0.37 - 2.31) | 0.45 | (0.00011) |
| 10 weeks | 1.38 | (0.75 - 2.55) | 0.84 | (0.35 - 2.05) | 0.45 | (0.00011) |
| 11 weeks | 1.44 | (0.78 - 2.64) | 0.84 | (0.35 - 2.05) | 0.43 | (0.00011) |
| 12 weeks | 1.44 | (0.78 - 2.64) | 0.71 | (0.30 - 1.67) | 0.43 | (0.00011) |
| Normal kidney function | | | | | | |
| 1 week | 1.33 | (0.40 - 4.38) | 0.66 | (0.16 - 2.77) | 0.06 | (0.00033) |
| 2 weeks | 0.80 | (0.32 - 2.00) | 0.66 | (0.16 - 2.77) | 0.78 | (0.00013) |
| 3 weeks | 0.95 | (0.45 - 2.03) | 0.66 | (0.16 - 2.77) | 0.59 | (0.00011) |
| 4 weeks | 0.95 | (0.45 - 2.03) | 0.88 | (0.23 - 3.30) | 0.59 | (0.00011) |
| 5 weeks | 1.18 | (0.59 - 2.37) | 0.88 | (0.23 - 3.30) | 0.52 | (0.00011) |
| 6 weeks | 1.24 | (0.64 - 2.38) | 0.92 | (0.28 - 3.03) | 0.60 | (0.00011) |
| 7 weeks | 1.24 | (0.64 - 2.38) | 0.94 | (0.31 - 2.83) | 0.60 | (0.00011) |
| 8 weeks | 1.30 | (0.68 - 2.49) | 0.96 | (0.35 - 2.68) | 0.58 | (0.00011) |
| 9 weeks | 1.37 | (0.72 - 2.60) | 0.77 | (0.29 - 2.03) | 0.53 | (0.00011) |
| 10 weeks | 1.36 | (0.73 - 2.53) | 0.69 | (0.27 - 1.81) | 0.53 | (0.00011) |
| 11 weeks | 1.42 | (0.76 - 2.64) | 0.69 | (0.27 - 1.81) | 0.50 | (0.00011) |
| 12 weeks | 1.42 | (0.76 - 2.64) | 0.63 | (0.25 - 1.63) | 0.50 | (0.00011) |
| Chronic kidney disease | | | | | | |
| 1 week | 0.65 | (0.013 - 33.31) | 0.65 | (0.013 - 33.31) | - | - |
| 2 weeks | 0.65 | (0.013 - 33.31) | 0.65 | (0.013 - 33.31) | - | - |
| 3 weeks | 0.65 | (0.013 - 33.31) | 0.65 | (0.013 - 33.31) | - | - |
| 4 weeks | 0.65 | (0.013 - 33.31) | 1.98 | (0.079 - 49.70) | >999.99 | (0.37) |
| 5 weeks | 0.65 | (0.013 - 33.31) | 1.98 | (0.079 - 49.70) | >999.99 | (0.37) |
| 6 weeks | 3.35 | (0.16 - 71.55) | 1.98 | (0.079 - 49.70) | >999.99 | (0.99) |
| 7 weeks | 3.35 | (0.16 - 71.55) | 1.98 | (0.079 - 49.70) | >999.99 | (0.99) |
| 8 weeks | 3.35 | (0.16 - 71.55) | 1.98 | (0.079 - 49.70) | >999.99 | (0.99) |
| 9 weeks | 3.35 | (0.16 - 71.55) | 3.35 | (0.16 - 71.55) | >999.99 | (0.99) |
| 10 weeks | 3.35 | (0.16 - 71.55) | 3.35 | (0.16 - 71.55) | >999.99 | (0.85) |
| 11 weeks | 3.35 | (0.16 - 71.55) | 3.35 | (0.16 - 71.55) | >999.99 | (0.85) |
| 12 weeks | 3.35 | (0.16 - 71.55) | 1.30 | (0.11 - 14.83) | 0.45 | (0.00018) |

OR, odds ratio; CI, confidence interval; SE, standard error.

**Table S4. Incidence of Drug-Induced Liver Injury in Renal Function Subgroups**

| | Total | | G/P | | EBR/GZR | | *p - value* | IRR | (95% CI) | *p - value* |
|---|---|---|---|---|---|---|---|---|---|---|
| | *n* | (%) | *n* | (%) | *n* | (%) | | | | |
| Overall | | | | | | | | | | |
| Event, *n* (%) | 22 | (2.63) | 9 | (2.18) | 13 | (3.06) | 0.43 | 0.71 | (0.31 - 1.67) | 0.44 |
| ALT >5×ULN | 12 | (1.43) | 4 | (0.97) | 8 | (1.88) | 0.27 | 0.52 | (0.16 - 1.71) | 0.28 |
| AST >5×ULN | 7 | (0.84) | 3 | (0.73) | 4 | (0.94) | 0.74 | 0.77 | (0.17 - 3.46) | 0.74 |
| ALT/AST >3×ULN +T-Bil >2×ULN† | 2 | (0.24) | 0 | (0.00) | 2 | (0.47) | 0.16 | - | - | - |
| ALT/AST >3×ULN + INR >1.5 | 2 | (0.24) | 1 | (0.24) | 1 | (0.24) | 0.98 | 1.03 | (0.06 - 16.49) | 0.98 |
| T-Bil >2×ULN + INR >1.5 | 2 | (0.24) | 1 | (0.24) | 1 | (0.24) | 0.98 | 1.03 | (0.06 - 16.49) | 0.98 |
| Normal kidney function | | | | | | | | | | |
| Event, *n* (%) | 19 | (2.59) | 7 | (2.00) | 12 | (3.12) | 0.34 | 0.64 | (0.25 - 1.63) | 0.35 |
| ALT >5×ULN | 11 | (1.50) | 4 | (1.14) | 7 | (1.82) | 0.45 | N0.63 | (0.18 - 2.15) | 0.46 |
| AST >5×ULN | 6 | (0.82) | 2 | (0.57) | 4 | (1.04) | 0.48 | 0.55 | (0.10 - 3.00) | 0.49 |
| ALT/AST >3×ULN +T-Bil >2×ULN† | 2 | (0.27) | 0 | (0.00) | 2 | (0.52) | 0.18 | - | - | - |
| ALT/AST >3×ULN + INR >1.5 | 1 | (0.14) | 0 | (0.00) | 1 | (0.26) | 0.34 | 1.10 | (0.07 - 17.59) | 0.95 |
| T-Bil >2×ULN + INR >1.5 | 2 | (0.27) | 1 | (0.29) | 1 | (0.26) | 0.95 | - | - | - |
| Chronic kidney disease | | | | | | | | | | |
| Event, *n* (%) | 3 | (2.94) | 2 | (3.23) | 1 | (2.50) | 0.83 | 1.29 | (0.12 - 14.23) | 0.84 |
| ALT >5×ULN | 1 | (0.98) | 0 | (0.00) | 1 | (2.50) | 0.21 | - | - | - |
| AST >5×ULN | 1 | (0.98) | 1 | (1.61) | 0 | (0.00) | 0.42 | - | - | - |
| ALT/AST >3×ULN +T-Bil >2×ULN† | - | - | - | - | - | - | - | - | - | - |
| ALT/AST >3×ULN + INR >1.5 | 1 | (0.98) | 1 | (1.61) | 0 | (0.00) | 0.42 | - | - | - |
| T-Bil >2×ULN + INR >1.5 | - | - | - | - | - | - | - | - | - | - |

EBR/GZR, elbasvir/grazoprevir; G/P, glecaprevir / pibrentasvir; ALT, alanine aminotransferase; AST, aspartate aminotransferase; T-Bil, total bilirubin; INR, international normalized ratio; IRR, incidence rate ratio; CI, confidence interval; ULN, upper limit of normal.
†Including the patients with ALT> 5 times ULN.

**Table S5. Sensitivity Analysis of ALT/AST Level Alterations Across Renal Function Subgroups**

| | Overall | | G/P | | EBR/GZR | | *p - value* | IRR | (95% CI) | *p - value* |
|---|---|---|---|---|---|---|---|---|---|---|
| | *n* | (%) | *n* | (%) | *n* | (%) | | | | |
| Overall | | | | | | | | | | |
| Event, *n* (%) | 47 | (5.62) | 23 | (5.58) | 24 | (5.65) | 0.97 | 0.99 | (0.56 - 1.75) | 0.97 |
| ALT >3×ULN | 30 | (3.58) | 14 | (3.40) | 16 | (3.76) | 0.78 | 0.90 | (0.44 - 1.85) | 0.78 |
| AST >3×ULN | 37 | (4.42) | 17 | (4.13) | 20 | (4.71) | 0.68 | 0.88 | (0.46 - 1.67) | 0.69 |
| ALT/AST >3×ULN +T-Bil >2×ULN† | 2 | (0.24) | 0 | (0.00) | 2 | (0.47) | 0.16 | - | - | - |
| ALT/AST >3×ULN + INR >1.5 | 2 | (0.24) | 1 | (0.24) | 1 | (0.24) | 0.98 | 1.03 | (0.06 - 16.49) | 0.98 |
| T-Bil >2×ULN + INR >1.5 | 2 | (0.24) | 1 | (0.24) | 1 | (0.24) | 0.98 | 1.03 | (0.06 - 16.49) | 0.98 |
| Normal kidney function | | | | | | | | | | |
| Event, *n* (%) | 44 | (5.99) | 21 | (6.00) | 23 | (5.97) | 0.99 | 1.00 | (0.56 - 1.81) | 0.99 |
| ALT >3×ULN | 28 | (3.81) | 13 | (3.71) | 15 | (3.90) | 0.90 | 0.95 | (0.45 - 2.00) | 0.90 |
| AST >3×ULN | 34 | (4.63) | 15 | (4.29) | 19 | (4.94) | 0.68 | 0.87 | (0.44 - 1.71) | 0.68 |
| ALT/AST >3×ULN +T-Bil >2×ULN† | 2 | (0.27) | 0 | (0.00) | 2 | (0.52) | 0.18 | - | - | - |
| ALT/AST >3×ULN + INR >1.5 | 1 | (0.14) | 0 | (0.00) | 1 | (0.26) | 0.34 | 1.10 | (0.07 - 17.59) | 0.95 |
| T-Bil >2×ULN + INR >1.5 | 2 | (0.27) | 1 | (0.29) | 1 | (0.26) | 0.95 | - | - | - |
| Chronic kidney disease | | | | | | | | | | |
| Event, *n* (%) | 3 | (2.94) | 2 | (3.23) | 1 | (2.50) | 0.83 | 1.29 | (0.12 - 14.23) | 0.84 |
| ALT >3×ULN | 2 | (1.96) | 1 | (1.61) | 1 | (2.50) | 0.75 | 0.65 | (0.04 - 10.32) | 0.76 |
| AST >3×ULN | 3 | (2.94) | 2 | (3.23) | 1 | (2.50) | 0.83 | 1.29 | (0.12 - 14.23) | 0.84 |
| ALT/AST >3×ULN +T-Bil >2×ULN† | - | - | - | - | - | - | - | - | - | - |
| ALT/AST >3×ULN + INR >1.5 | 1 | (0.98) | 1 | (1.61) | 0 | (0.00) | 0.42 | - | - | - |
| T-Bil >2×ULN + INR >1.5 | - | - | - | - | - | - | - | - | - | - |

EBR/GZR, elbasvir/grazoprevir; G/P, glecaprevir / pibrentasvir; ALT, alanine aminotransferase; AST, aspartate aminotransferase; T-Bil, total bilirubin; INR, international normalized ratio; IFN, interferon; RBV, ribavirin; IRR, incidence rate ratio; CI, confidence interval; ULN, upper limit of normal.
†Including the patients with ALT> 5 times ULN.

**Table S6. The patients of DILI event characteristics**

| | Overall | | G/P | | EBR/GZR | | IRR | (95% CI) | P-value |
|---|---|---|---|---|---|---|---|---|---|
| | N | (%) | N | (%) | N | (%) | | | |
| Event patients | 22 | (100.00) | 9 | (40.91) | 13 | (59.09) | | | |
| Viral response | | | | | | | | | |
| SVR | 20/22 | (90.91) | 9/9 | (100.00) | 11/13 | (84.62) | | | |
| Virologic Failure | 0 | (0.00) | 0 | (0.00) | 0 | (0.00) | | | |
| Relapse | 0 | (0.00) | 0 | (0.00) | 0 | (0.00) | | | |
| Lose follow-up | 2 | (9.09) | 0 | (0.00) | 2 | (15.38) | | | |
| The patients in CKD stage | | | | | | | | | |
| Stage1 | 13 | (59.09) | 3 | (33.33) | 10 | (76.92) | | | |
| Stage 2 (mild) | 6 | (27.27) | 4 | (44.44) | 2 | (15.38) | | | |
| Stage 3a (moderate) | 1 | (4.55) | 0 | (0.00) | 1 | (7.69) | | | |
| Stage 3b (moderate) | 1 | (4.55) | 1 | (11.11) | 0 | (0.00) | | | |
| Stage 4 (severe) | - | - | - | - | - | - | | | |
| Stage 5 | 1 | (4.55) | 1 | (11.11) | 0 | (0.00) | | | |
| Age groups | | | | | | | | | |
| Overall | 22 | (100.00) | 9 | (40.91) | 13 | (59.09) | - | - | - |
| >75 years | 6 | (27.27) | 3 | (33.33) | 3 | (23.08) | - | - | - |
| 65-74 years | 6 | (27.27) | 2 | (22.22) | 4 | (30.77) | - | - | - |
| 55-64 years | 6 | (27.27) | 2 | (22.22) | 4 | (30.77) | 1.92 | (0.17 - 21.17) | 0.59 |
| 40-54 years | 3 | (13.64) | 1 | (11.11) | 2 | (15.38) | 0.46 | (0.04 - 5.04) | 0.52 |
| 20-39 years | 1 | (4.55) | 1 | (11.11) | 0 | (0.00) | - | - | - |
| <20 years | - | - | - | - | - | - | - | - | - |
| Normal kidney function | 19 | (100.00) | 7 | (36.84) | 12 | (63.16) | - | - | - |
| >75 years | 5 | (26.32) | 2 | (28.57) | 3 | (25.00) | - | - | - |
| 65-74 years | 4 | (21.05) | 1 | (14.29) | 3 | (25.00) | - | - | - |
| 55-64 years | 6 | (31.58) | 2 | (28.57) | 4 | (33.33) | 2.02 | (0.18 - 22.26) | 0.57 |
| 40-54 years | 3 | (15.79) | 1 | (14.29) | 2 | (16.67) | 0.47 | (0.04 - 5.20) | 0.54 |
| 20-39 years | 1 | (5.26) | 1 | (14.29) | 0 | (0.00) | - | - | - |
| <20 years | - | - | - | - | - | - | - | - | - |
| Chronic kidney disease | 3 | (100.00) | 2 | (66.67) | 1 | (33.33) | - | - | - |
| >75 years | 1 | (33.33) | 1 | (50.00) | 0 | (0.00) | - | - | - |
| 65-74 years | 2 | (66.67) | 1 | (50.00) | 1 | (100.00) | - | - | - |
| 55-64 years | - | - | - | - | - | - | - | - | - |
| 40-54 years | - | - | - | - | - | - | - | - | - |
| 20-39 years | - | - | - | - | - | - | - | - | - |

| | | | | | | | | | |
|---|---|---|---|---|---|---|---|---|---|
| <20 years | - | - | - | - | - | - | - | - | - |
| BMI range | | | | | | | | | |
| Overall | 22 | (100.00) | 9 | (40.91) | 13 | (59.09) | - | - | - |
| >30 | - | (-) | - | (-) | - | (-) | - | - | - |
| 25-29 | 7 | (31.82) | 3 | (42.86) | 4 | (30.77) | 0.78 | (0.07 - 8.62) | 0.84 |
| 18.5-24 | 4 | (18.18) | 1 | (25) | 3 | (23.08) | - | - | - |
| <18.5 | 11 | (50.00) | 5 | (45.45) | 6 | (46.15) | 0.93 | (0.19 - 4.60) | 0.93 |
| Normal kidney function | 19 | (100.00) | 7 | (36.84) | 12 | (63.16) | - | - | - |
| >30 | - | - | - | - | - | - | - | - | - |
| 25-29 | 7 | (36.84) | 3 | (42.86) | 4 | (33.33) | 0.88 | (0.08 - 9.76) | 0.92 |
| 18.5-24 | 3 | (15.79) | 0 | (0.00) | 3 | (25.00) | - | - | - |
| <18.5 | 9 | (47.37) | 4 | (57.14) | 5 | (41.67) | 0.96 | (0.19 - 4.75) | 0.96 |
| Chronic kidney disease | 3 | (100.00) | 2 | (66.67) | 1 | (33.33) | - | - | - |
| >30 | - | - | - | - | - | - | - | - | - |
| 25-29 | - | - | - | - | - | - | - | - | - |
| 18.5-24 | 1 | (33.33) | 1 | (50.00) | 0 | (0.00) | - | - | - |
| <18.5 | 2 | (66.67) | 1 | (50.00) | 1 | (100.00) | - | - | - |

EBR/GZR, elbasvir/grazoprevir; G/P, glecaprevir / pibrentasvir; CKD, Chronic kidney disease; BMI, Body Mass Index

**Table S7. Evaluate the DILI event risk of comorbidities and co-administered drug**

| | Event (N=22) | | Crude | | Adjusted | | P-value |
|---|---|---|---|---|---|---|---|
| | N | (%) | OR | (95% CI) | OR | (95% CI) | |
| Comorbidities | | | | | | | |
| Hepatic fibrosis | 4 | (4.71) | 2.01 | (0.67 - 6.10) | 0.49 | (0.12 - 2.11) | 0.34 |
| Liver tumor | 9 | (18.75) | 13.78 | (5.55 - 34.18) | 18.89 | (5.40 - 66.12) | <0.001* |
| Cirrhosis | 0 | (0.00) | <0.001 | (<0.001 - >999.99) | <0.001 | (<0.001 - >999.99) | 0.99 |
| Digestive system neoplasms | 4 | (3.13) | 1.24 | (0.41 - 3.72) | 0.94 | (0.25 - 3.55) | 0.92 |
| T2DM | 0 | (0.00) | <0.001 | (<0.001 - >999.99) | <0.001 | (<0.001 - >999.99) | 0.85 |
| Hyperlipidaemia | 1 | (0.99) | 0.34 | (0.045 - 2.56) | 0.01 | (<0.001 - >999.99) | 0.99 |
| Hypertension | 11 | (5.07) | 2.96 | (1.26 - 6.92) | 4.25 | (1.49 - 12.15) | 0.01* |
| Peptic ulcer | 6 | (2.06) | 0.70 | (0.27 - 1.80) | 0.73 | (0.21 - 2.46) | 0.61 |
| Constipation | 3 | (2.08) | 0.76 | (0.22 - 2.59) | 0.81 | (0.19 - 3.50) | 0.78 |
| Dizziness and giddiness | 1 | (4.55) | 0.23 | (0.03 - 1.72) | 0.13 | (0.01 - 1.36) | 0.09 |
| Functional dyspepsia | 1 | (0.83) | 0.28 | (0.037 - 2.09) | 0.30 | (0.03 - 2.99) | 0.30 |
| GERD with esophagitis | 3 | (2.26) | 0.83 | (0.24 - 2.85) | 1.18 | (0.27 - 5.08) | 0.82 |
| Sleep disorder | 3 | (4.55) | 1.31 | (0.38 - 4.50) | 1.68 | (0.38 - 7.56) | 0.50 |
| UTI | 2 | (1.63) | 0.57 | (0.13 - 2.49) | 0.49 | (0.10 - 2.52) | 0.39 |
| Co-administered drug | | | | | | | |
| Famotidine 20 mg | 9 | (3.9) | 1.85 | (0.78 - 4.39) | 1.36 | (0.44 - 4.20) | 0.60 |
| Proheparum tab | 4 | (6.06) | 2.70 | (0.89 - 8.22) | 2.45 | (0.65 - 9.28) | 0.19 |
| Pantoprazole 40 mg | 0 | (0.00) | <0.001 | (<0.001 - >999.99) | <0.001 | (<0.001 - >999.99) | 0.67 |
| Mosapride citrate 5 mg | 3 | (2.73) | 1.05 | (0.30 - 3.59) | 1.64 | (0.33 - 8.22) | 0.55 |
| Sennosides 20 mg | 4 | (3.85) | 1.59 | (0.53 - 4.79) | 0.93 | (0.20 - 4.45) | 0.93 |

OR: Odds ratio; T2DM, Type 2 diabetes mellitus; GERD, Gastroesophageal reflux disease; UTI, Urinary Tract Infection
*Significant difference ($p < 0.05$).

***Treatment effectiveness***

The effectiveness outcome was the achievement of SVR12 in the intention-to-treat (ITT) population, which comprised all patients treated with EBR/GZR or G/P for whom data was available on SVR12, virological failure, or relapse, or who were lost to follow-up; and in the per-protocol (PP) population, which comprised the ITT population, except those lost to follow-up.

Overall, SVR12 was achieved in 92.71% (95%CI, 90.88–94.54). In the EBR/GZR and G/P groups, 86.35% (95%CI, 83.09–89.61) and 99.27% (95%CI, 98.45–100.1) achieved SVR12, respectively. There was curative failure in one EBR/GZR patient. Relapse occurred in 7/837 (0.84%), of whom, six had received EBR/GZR and one had received G/P. Of the 53 patients lost to follow-up, 51 were in the EBR/GZR group, and two were in the G/P group.

The SVR12 rates of the NFK subgroups were as follows: 92.93% (95%CI, 91.04–94.76) in the ITT population, 98.84% (95%CI, 98.04–99.64) in the PP population. In the ITT and PP EBR/GZR patients, the SVR12 were 87.01% (95%CI, 83.64–90.36) and 97.95% (95%CI, 96.45–99.45), respectively. In the ITT and PP G/P patients, the SVR12 rates were 99.43% (95%CI, 98.59–100.21) and 99.71% (95%CI, 99.15–100.27), respectively. The SVR12 rates of the ITT and PP populations in the whole CKD group were 91.18% (95%CI, 85.7–96.7) and 100%, respectively. In the ITT and PP EBR/GZR CKD subgroups, they were 80% (95%CI, 67.6–92.4) and 100%, respectively. In the ITT and PP G/P CKD subgroups, they were 98.39% (95%CI, 95.28–101.52) and 100%, respectively.

**Table S8. Population in effectiveness analysis (ITT).**

| Characteristics | Overall | | | G/P | | | EBR/GZR | | | p-value |
|---|---|---|---|---|---|---|---|---|---|---|
| | N | % | (95% CI) | N | % | (95% CI) | N | % | (95% CI) | |
| Overall | | | | | | | | | | |
| SVR | 776 | 92.71 | (90.88 - 94.54) | 409 | 99.27 | (98.45 - 100.10) | 367 | 86.35 | (83.09 - 89.61) | <0.001* |
| Virologic Failure | 1 | 0.12 | (-0.12 - 0.36) | 0 | 0.00 | (0 - 0) | 1 | 0.24 | (-0.23 - 0.71) | 0.32 |
| Relapse | 7 | 0.84 | (0.20 - 1.48) | 1 | 0.24 | (0.23 - 0.71) | 6 | 1.41 | (0.29 - 2.53) | 0.06 |
| Lose follow-up | 53 | 6.33 | (4.68 - 7.98) | 2 | 0.49 | (-0.19 - 1.16) | 51 | 12 | (8.91 - 15.09) | <0.001* |
| Normal kidney function | | | | | | | | | | |
| SVR | 683 | 92.93 | (91.04 - 94.76) | 348 | 99.43 | (98.59 - 100.21) | 335 | 87.01 | (83.64 - 90.36) | <0.001* |
| Virologic Failure | 1 | 0.14 | (-0.13 - 0.33) | 0 | 0.00 | (0 - 0) | 1 | 0.26 | (-0.25 - 0.77) | 0.34 |
| Relapse | 7 | 0.95 | (0.28 - 1.72) | 1 | 0.29 | (-0.27 - 0.85) | 6 | 1.56 | (0.35 - 2.85) | 0.08 |
| Lose follow-up | 44 | 5.99 | (4.28 - 7.72) | 1 | 0.29 | (-0.27 - 0.85) | 43 | 11.17 | (8.05 - 14.35) | <0.001* |
| Chronic kidney disease | | | | | | | | | | |
| SVR | 93 | 91.18 | (85.70 - 96.70) | 61 | 98.39 | (95.28 - 101.52) | 32 | 80 | (67.60 - 92.40) | 0.01* |
| Virologic Failure | 0 | 0.00 | (0 - 0) | 0 | 0.00 | (0 - 0) | 0 | 0.00 | (0 - 0) | 0 |
| Relapse | 0 | 0.00 | (0 - 0) | 0 | 0.00 | (0 - 0) | 0 | 0.00 | (0 - 0) | 0 |
| Lose follow-up | 9 | 8.82 | (3.30 - 14.30) | 1 | 1.61 | (-1.52 - 4.72) | 8 | 20 | (7.60 - 32.4) | 0.01* |

EBR/GZR, elbasvir/grazoprevir; G/P, glecaprevir / pibrentasvir; SVR, sustained virological response.
*Significant difference ($p < 0.05$).

**Table S9. Successful cure rate for different categories at baseline (ITT).**

| Characteristics | Overall | | G/P | | EBR/GZR | | p-value |
|---|---|---|---|---|---|---|---|
| | N | (%) | N | (%) | N | (%) | |
| Gender | | | | | | | |
| Male | 331/361 | (91.69) | 172/175 | (98.29) | 159/186 | (85.48) | <0.001* |
| Female | 445/476 | (93.49) | 237/237 | (100.00) | 208/239 | (87.03) | <0.001* |
| HCV genotype | | | | | | | |
| GT1a | 10/10 | (100.00) | 9/9 | (100.00) | 1/1 | (100.00) | - |
| GT1b | 299/326 | (91.72) | 78/78 | (100.00) | 221/248 | (89.11) | 0.01* |
| GT12 | 3/3 | (100.00) | 3/3 | (100.00) | 0/0 | (0.00) | - |
| undetected | 467/501 | (93.21) | 322/325 | (99.08) | 145/176 | (82.39) | <0.001* |
| Therapy duration at weeks | | | | | | | |
| 8 | 591/611 | (96.73) | 391/393 | (99.49) | 200/218 | (91.74) | <0.001* |
| 12 | 185/226 | (81.86) | 18/19 | (94.74) | 167/207 | (80.68) | 0.13 |
| Peritoneal Dialysis | 10/10 | (100.00) | 8/8 | (100.00) | 2/2 | (100.00) | - |
| The patients in CKD stage | | | | | | | |
| Stage1 | 500/543 | (92.08) | 263/265 | (99.25) | 237/278 | (85.25) | <0.001* |
| Stage 2 (mild) | 183/192 | (95.31) | 85/85 | (100.00) | 98/107 | (91.59) | 0.01* |
| Stage 3a (moderate) | 22/24 | (91.67) | 14/14 | (100.00) | 8/25.18 | (80.00) | 0.08 |
| Stage 3b (moderate) | 10/12 | (83.33) | 6/6 | (100.00) | 4/6 | (66.67) | 0.12 |
| Stage 4 (severe) | 11/12 | (91.67) | 6/6 | (100.00) | 5/6 | (83.33) | 0.30 |
| Stage 5 | 50/54 | (92.59) | 35/36 | (97.22) | 15/18 | (83.33) | 0.07 |
| Age groups | | | | | | | |
| >75 years | 117/129 | (90.70) | 51/52 | (98.08) | 66/77 | (85.71) | 0.02* |
| 65-74 years | 169/184 | (91.85) | 77/77 | (100.00) | 92/107 | (85.98) | 0.01* |
| 55-64 years | 225/245 | (91.84) | 124/125 | (99.20) | 101/120 | (84.17) | <0.001* |
| 40-54 years | 209/222 | (94.14) | 116/116 | (100.00) | 93/106 | (87.74) | <0.001* |
| 20-39 years | 55/56 | (98.21) | 40/41 | (97.56) | 15/15 | (100.00) | 0.54 |
| <20 years | 1/1 | (100.00) | 1/1 | (100.00) | 0/0 | (0.00) | - |
| BMI range | | | | | | | |
| >30 | 18/20 | (90.00) | 9/9 | (100.00) | 9/11 | (81.82) | 0.18 |
| 25-29 | 72/82 | (87.80) | 32/32 | (100.00) | 40/50 | (80.00) | 0.01* |
| 18.5-24 | 122/135 | (90.37) | 60/60 | (100.00) | 62/75 | (82.67) | 0.01* |
| <18.5 | 564/600 | (94.00) | 308/311 | (99.04) | 256/289 | (88.58) | <0.001* |
| Comorbidities | | | | | | | |

| | | | | | | | |
|---|---|---|---|---|---|---|---|
| Cirrhosis | 14/15 | (93.33) | 5/5 | (100.00) | 9/10 | (90.00) | 0.46 |
| Hepatic fibrosis | 70/85 | (82.35) | 32/32 | (100.00) | 38/53 | (71.70) | 0.01* |
| Peritoneal Dialysis | 10/10 | (100.00) | 8/8 | (100.00) | 2/2 | (100.00) | - |
| Liver tumor | 41/48 | (85.42) | 15/15 | (100.00) | 26/33 | (78.79) | 0.05 |
| Digestive system neoplasms | 111/128 | (86.72) | 47/47 | (100.00) | 64/81 | (79.01) | 0.01* |
| Type 2 diabetes mellitus | 12/17 | (70.59) | 3/3 | (100.00) | 9/14 | (64.29) | 0.22 |
| Hyperlipidaemia | 87/101 | (86.14) | 40/40 | (100.00) | 47/61 | (77.05) | 0.01* |
| Hypertension | 192/217 | (88.48) | 90/90 | (100.00) | 102/127 | (80.31) | <0.001* |
| Peptic ulcer | 258/291 | (88.66) | 118/118 | (100.00) | 140/173 | (80.92) | <0.001* |
| Gastric ulcer | 224/247 | (90.69) | 113/114 | (99.12) | 111/133 | (83.46) | <0.001* |
| Constipation | 124/144 | (86.11) | 57/58 | (98.28) | 67/86 | (77.91) | 0.01* |
| Dizziness and giddiness | 130/142 | (91.55) | 61/61 | (100.00) | 69/81 | (85.19) | 0.01* |
| Functional dyspepsia | 105/120 | (87.50) | 42/42 | (100.00) | 63/78 | (80.77) | 0.01* |
| GERD with esophagitis | 118/133 | (88.72) | 52/52 | (100.00) | 66/81 | (81.48) | 0.01* |
| Acute abdomen | 115/128 | (89.84) | 58/58 | (100.00) | 57/70 | (81.43) | 0.01* |
| Urinary Tract Infection | 108/123 | (87.80) | 48/48 | (100.00) | 60/75 | (80.00) | 0.01* |
| Sleep disorder | 84/91 | (92.31) | 41/41 | (100.00) | 43/50 | (86.00) | 0.01* |
| Anxiety disorder | 67/78 | (85.90) | 31/33 | (93.94) | 36/45 | (80.00) | 0.08 |
| peptic ulcer with hemorrhage | 48/59 | (81.36) | 24/24 | (100.00) | 24/35 | (68.57) | 0.01* |
| Irritable bowel syndrome | 129/143 | (90.21) | 67/67 | (100.00) | 62/76 | (81.58) | <0.001* |
| Flatulence | 38/43 | (88.37) | 18/18 | (100.00) | 20/25 | (80.00) | 0.04* |

EBR/GZR, elbasvir/grazoprevir; G/P, glecaprevir / pibrentasvir; *Significant difference ($p < 0.05$).

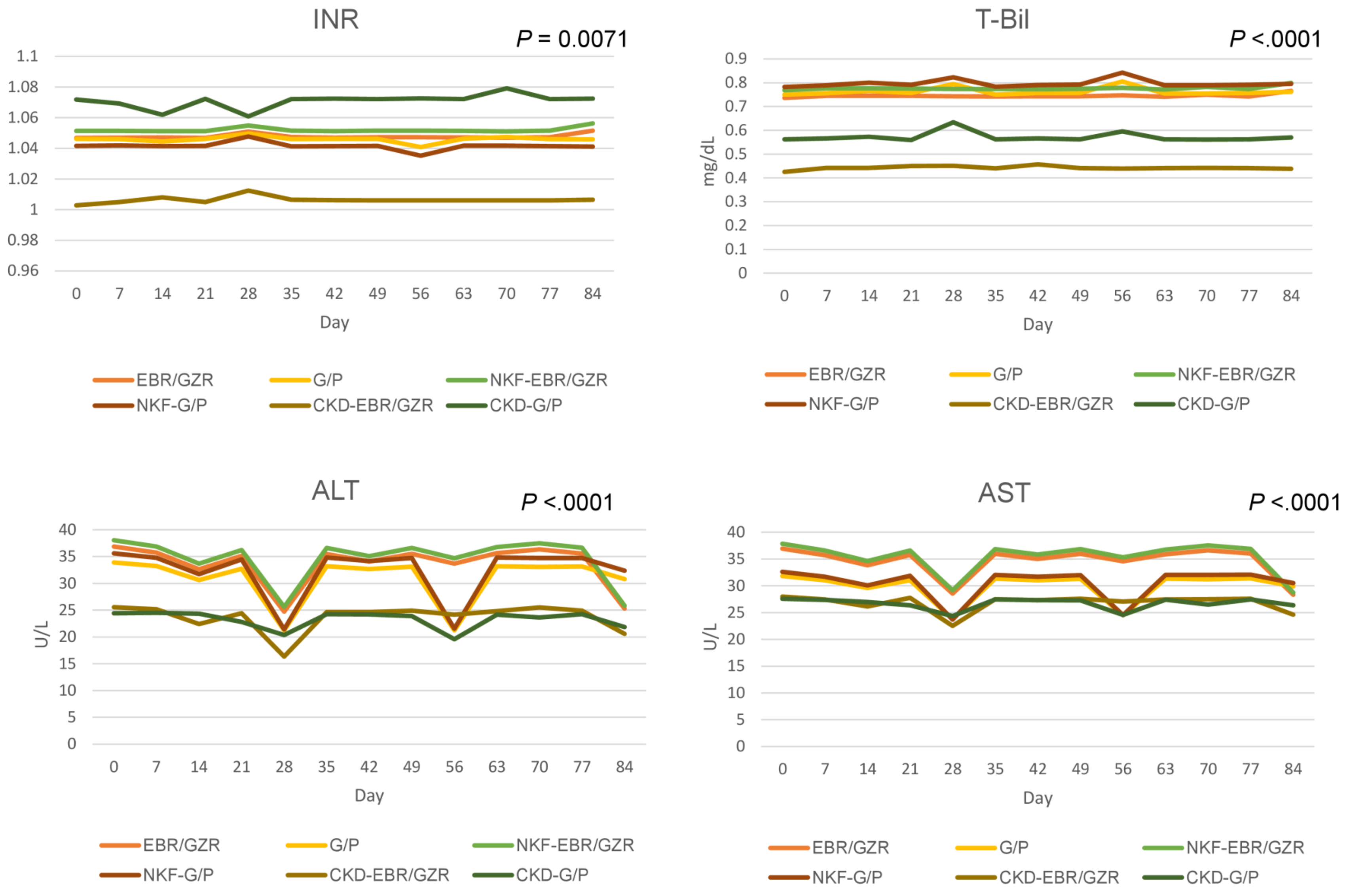


**Figure S1. Laboratory assessments.**
